# Performance Evaluation of Thermoelectric Energy Harvesting System on Operating Rolling Stock

**DOI:** 10.3390/mi9070359

**Published:** 2018-07-20

**Authors:** Dahoon Ahn, Kyungwho Choi

**Affiliations:** 1 Advanced Railroad Vehicle Division, Korea Railroad Research Institute, Uiwang-si 16105, Korea; dhahn@krri.re.kr; 2 New Transportation Innovative Research Center, Korea Railroad Research Institute, Uiwang-si 16105, Korea

**Keywords:** energy harvesting, thermoelectric generator, railroad vehicle, rolling stock, axle bearing

## Abstract

During rolling stock operation, various kinds of energy such as vibration, heat, and train-induced wind are dissipated. The amount of energy dissipation cannot be overlooked when a heavy railroad vehicle operates at high speed. Therefore, if the wasted energy is effectively harvested, it can be used to power components like low power sensor nodes. This study aims to review a method of collecting waste heat, caused by the axle bearing of bogie in a rolling stock. A thermoelectric module (TEM) was used to convert the temperature gradient between the surface of the axle bearing housing and the outdoor air into electric energy. In this study, the output performance by temperature difference in the TEM was lab-tested and maximized by computational fluid analysis of the cooling fins. The optimized thermoelectric energy harvesting system (TEHS) was designed and applied on a rolling stock to analyze the power-generating performance under operation. When the rolling stock was operated for approximately 57 min including an interval of maximum speed of 300 km/h, the maximum open circuit voltage was measured at approximately 0.4 V. Based on this study, the system is expected to be utilized as a self-powered independent monitoring system if applied to a low-power sensor node in the future.

## 1. Introduction

The railway system was a symbol of modernization and has been developed for more than 100 years with its advantages of mass transportation, punctuality, and fast speed. Currently, the advancement of the high-speed train has led to a new era of the railway system as a fast and safe form of public transportation [1,2,3,4]. For the safety of passengers within a rapid railway system, not only the reliability of the railway system but also the monitoring technology for understanding abnormal conditions must be improved. The KTX (high-speed railroad system in Korea), for instance, experiences time delays mainly because defects have occurred in the axles and vehicle wheels, which might result in serious problems related to derailments [5,6]. To solve the problem, railway monitoring systems detect and analyze abnormal temperature rises according to axle bearing degradation under operation, with an infrared sensor called the Hot Box Detector (HBD) installed at constant track intervals (approximately 40 km) [7]. Note, however, that errors may occur as fast-moving vehicles are measured with a remotely installed infrared light on the ground; moreover, real-time monitoring is impossible, so it is difficult to respond quickly when an abnormal situation occurs. Therefore, it is necessary to develop an on-board real-time monitoring system for axle bearing inspections. This system must be supplied with power, and this may incur installation costs for electric wires and maintenance costs. As such, harvesting many different kinds of waste energy generated by running vehicles may be the solution to these problems [4,8,9,10,11,12,13,14,15,16,17,18,19,20,21,22,23,24,25,26,27,28,29,30,31,32]. This study focused on the axle bearing housing, which is the boundary of high and low temperatures induced by heat conduction from inside the housing due to axle rotation and by cooling from outside due to the forced convection caused by the running vehicle. When a temperature gradient occurs constantly while in operation, waste heat can be collected due to the thermoelectric effect [33,34,35]. As the direct conversion of electric energy and heat energy, the thermoelectric effect can be classified into two: the Seebeck effect that converts heat energy into electric energy, and the Peltier effect that converts electric energy into heat energy. The Seebeck effect generates power through the difference in electric potential induced by the diffusion movement of the electron carriers. Therefore, installing thermoelectric module (TEM) on the axle bearing housings of boundary surfaces with a temperature difference and maximizing this temperature difference may be effective in generating power. In this study, a modularized thermoelectric energy harvesting system (TEHS) was developed by choosing a suitable TEM through performance test in the lab and optimizing the design of cooling fin in order to keep the given temperature difference as large as possible by utilizing the Seebeck effect. The system was then installed on a Korean high-speed train to analyze the output performance according to the driving condition, and we reviewed whether or not the driving power of the real-time monitoring system can be applied.

## 2. System Design

**Figure 1:**
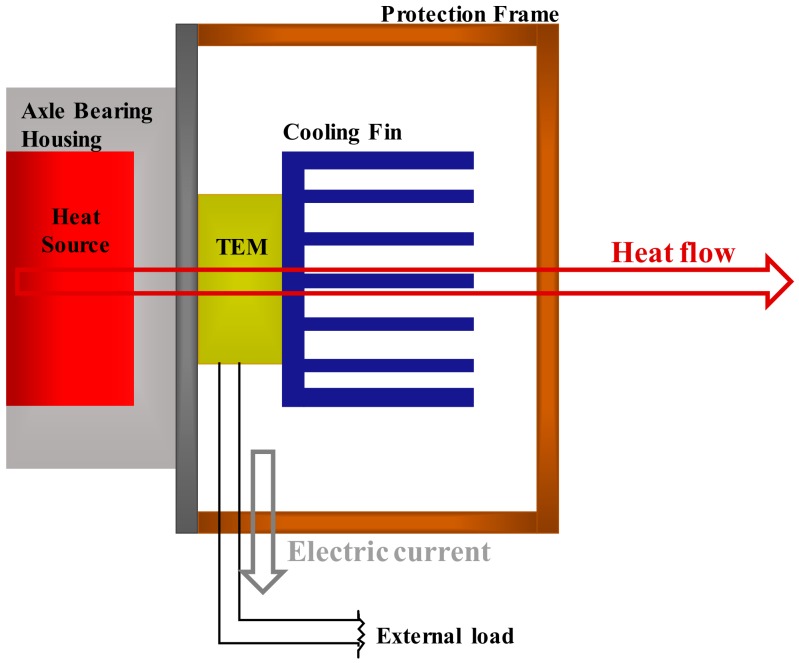
System architecture of thermoelectric energy harvesting system.

A TEHS consists of a TEM and a cooling system. As shown in Figure 1, one side of the TEM is attached to an axle bearing housing as the heat source, generating power by thermoelectric effect. A cooling system attached to the other side of the TEM improves the power-generating amount by increasing the temperature gradient on both sides of the TEM through effective cooling progress. In addition, the cooling system acts to protect the TEM from impact from external substances as the rolling stock runs fast and also serves as an installation frame fixing the system on the housing.

### 2.1. Thermoelectric Module

Two bulk types of TEM (128A1030, Peltron GmbH., Fürth, Germany and TK-1-3-S, HTRD, Anyang, Republic of Korea) were tested for thermoelectric energy harvesting on the axle bearing housing of the rolling stock. Both TEMs have 20 × 20 mm^2^ surface area as the maximum space where the TEM can be installed on the surface of the axle bearing housing. The temperature difference between the axle bearing housing—the high-temperature part while the train vehicle is running—and the outdoor air is approximately 15 °C [4], and the average operation temperature of the TEM is around room temperature; thus, according to this, a generally used commercial TEM optimized at room temperature was selected.

### 2.2. Cooling System

**Figure 2:**
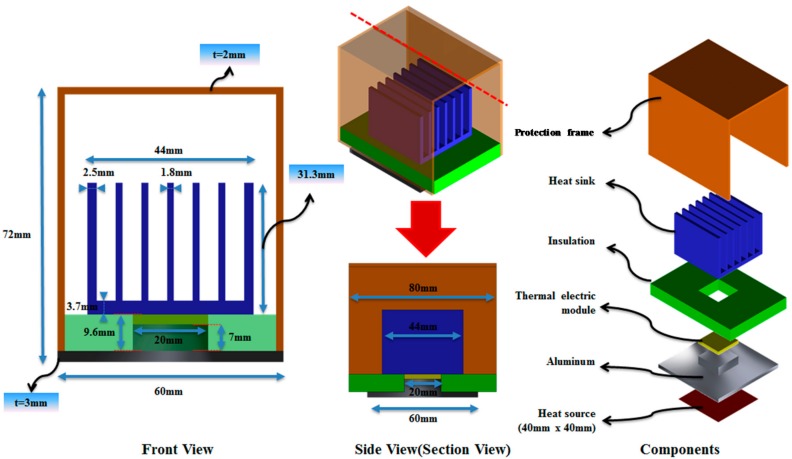
Illustration of the thermoelectric energy harvesting system (TEHS) for application on axle housing of rolling stock.

**Figure 3:**
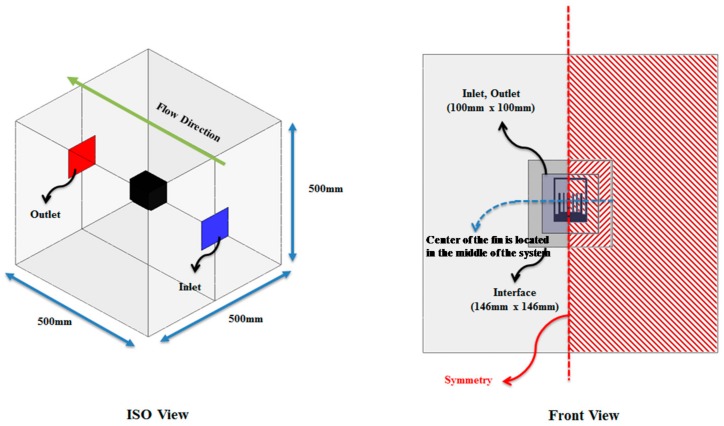
Boundary conditions of fluid region for numerical analysis.

In this study, the heat flux due to the temperature difference between the axle bearing housing of the rolling stock and the outdoor air was converted into electric energy by using a TEM. The thermal energy generated from the axle bearing as the heat source is dependent on the driving conditions of the railroad vehicle. As such, to improve the performance, this study attempted to maximize the air cooling effect by the driving of rolling stocks. Therefore, cooling fins were applied to the TEM, and numerical analysis on heat transfer through the air cooling of the TEM was performed to optimize the shape of the cooling fin. The analysis model is illustrated in Figure 2 and Figure 3. Installed at the bottom part of the TEM are an aluminum plate replicating the axle bearing housing and a size of 40 mm × 40 mm of an electric heater as a heat source.

To prevent unwanted thermal leakage, a thermal insulator is located around the TEM; installed on its upper part are the cooling fins to optimize the air cooling effect. On top of the TEM is a protection frame to protect the cooling fins and the TEM from the impact of ballast flying and substances occurring while driving. In Figure 3, the numerical analysis considers the flow interference and other types of effect by the cooling fins and protection frame; to shorten the calculating time, a bilateral symmetric model and 1-dimensional heat flux were assumed. With the numerical analysis, the temperature gradient of the high- and low-temperature parts of the TEM by changing the number and height of the plate-type cooling fin was analyzed, and the cooling fin shape was optimized to increase the power-generating performance as well.

**Figure 4:**
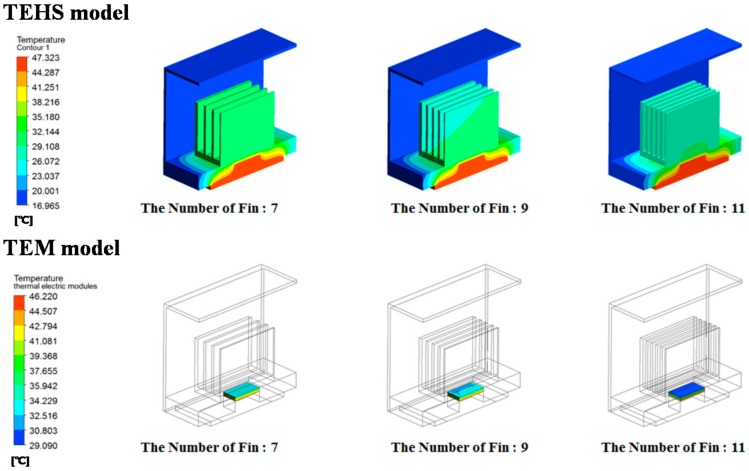
Numerical analysis results of temperature gradient of the TEHS with varying number of cooling fins.

**Figure 5:**
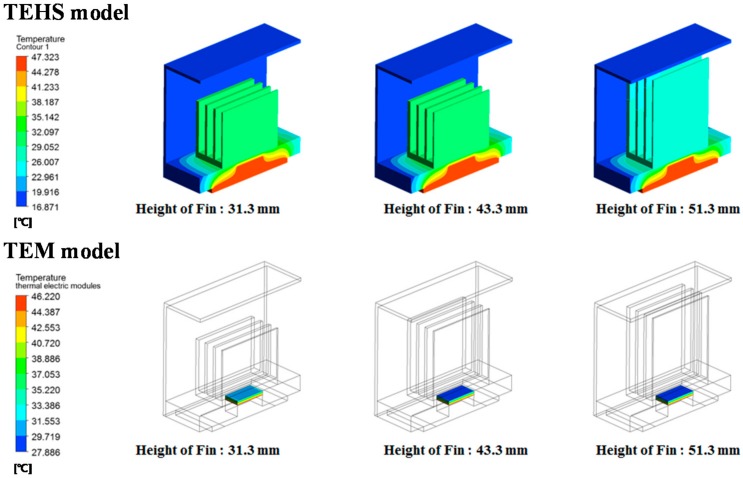
Numerical analysis results of temperature gradient of the TEHS with varying height of cooling fin.

As a result, the speed of the slipstream decreases as the number of cooling fins increases, resulting in flow resistance. Therefore, although the average surface heat transfer coefficient decreases, cooling performance improves by the increase of effective surface contact with air for heat transfer. By reviewing the temperature distribution of the entire system according to the number of cooling fins (Figure 4), the temperature of the low-temperature surface of the heatsink and the TEM decreases, whereas the temperature of the high-temperature surface is constant as the number of cooling fins increases. The configuration of the cooling fin at which its number is over 11 is not considered because there is a limitation regarding manufacturing thin configurations. According to the numerical analysis result by the change of height of the cooling fin (Figure 5), the speed of air passing through the fins decreases due to the interference effect of the fins and protection frame as the height of the fin increases. This may result in a decrease of heat dissipation into the air from the cooling fins; as with the analysis on the numbers of the cooling fin, however, the total heat flux increased due to the increase of the heat transfer area. Thus, a larger temperature gradient was formed on both sides of the TEM. A cooling fin height of more than 51.3 mm was not considered due to the interference of the protection frame. The size of the protection frame was determined by the regulation of vehicle limitation restricting the outermost size considering the attachment to the rolling stock.

**Figure 6:**
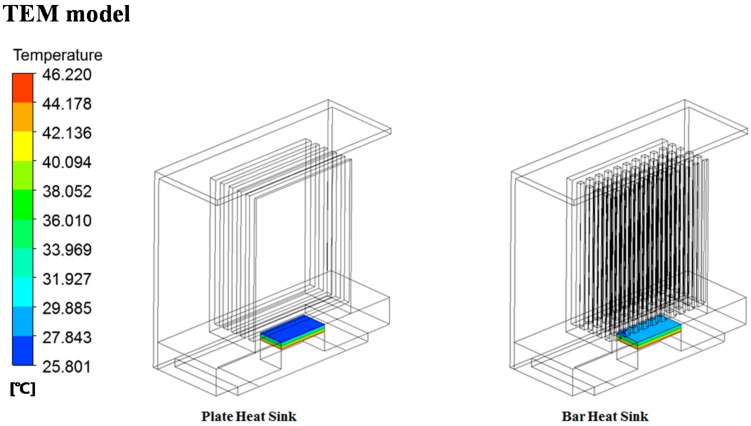
Numerical analysis results of temperature gradient of the thermoelectric module (TEM) with plate and bar type cooling fins.

The results of testing by two conditions are shown in Table 1. As the number and height of the fin shape limited by the size of the protection frame increase, the temperature gradient on both sides of the TEM increases. Generally, an air cooling system using cooling fins shows decreasing heat dissipation after reaching the optimized number and height of the fins [36]. Note, however, that the TEHS of this study does not see this kind of result due to the limit of increase in the number and height of the fins. Therefore, a numerical analysis was performed on a bar-type cooling fin that can increase the contact surface area where heat transfer occurs (Figure 6). Bar-type fins with a height of 51.3 mm, which showed optimal results in plate-type cooling fins, were fabricated and arranged at equal intervals in the length and width directions. All the analysis conditions except those mentioned above are the same as the plate-type cooling fins.

As a result, the TEHS shows a larger TEM temperate gradient for plate-type cooling fins than bar-type cooling fins. With the characteristics of the shape of the bar-type fins, the bar-type fins have strong turbulent flow between bars resulting, in an increased flow resistance; thus generating high pressure on the inhalation part of the fin. This pressure interrupts the flow inhaled into the system, so the flux flowing in the bar-type cooling fin is 50% of the plate-type cooling fin, and the total heat dissipation decreases. The effective surface on the bar-type cooling fin where the fin comes in contact with air is approximately 42% higher than the plate-type cooling fin, but heat transfer rarely occurs on the back side of the bar-type cooling fin due to the fluid congested between bars. Thus, the available surface area capable of heat transfer somewhat decreases. Based on the analysis result, a cooling system for applying the TEHS on rolling stock was designed.

## 3. Performance Test on TEMs

**Figure 7:**
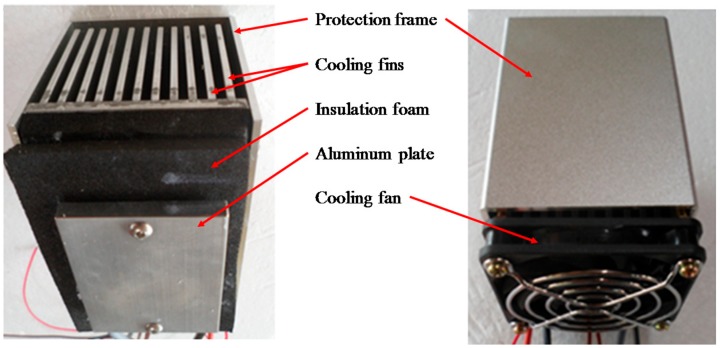
TEHS made for lab test based on the numerical analysis.

A TEHS replicating the condition of running rolling stock was fabricated utilizing a commercial TEM integrated with optimized cooling fins in order to test the output performance of the TEM, as shown in Figure 7. A thermocouple was installed in a 40 mm × 40 mm × 10 mm aluminum plate to measure the temperature of the high-temperature side of the TEM. Attached on one side of the aluminum plate was an additional TEM (HT-15-15-Lq 173, HTRD, Anyang, Korea) as heat source; on its other side, another TEM was installed for power generation. Placed on the upper side of the TEM were cooling fins, with a thermocouple mounted to measure the temperature of the low-temperature side of the TEM. A test model was designed with a cooling fan to simulate the cooling conditions by train-induced wind. The temperature difference between the low- and high-temperature sides of the TEM was controlled within 10–30 °C, similar to the temperature difference between the axle bearing housing and outdoor air when the rolling stock is under operation [4]. The output voltage and output current of the TEM were measured by varying the external load resistance connected to the TEM.

**Figure 8:**
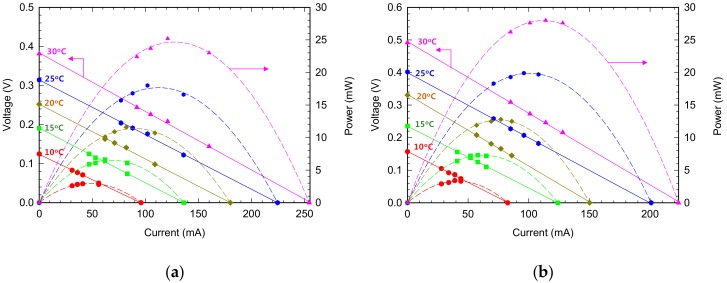
I–V characteristics of (**a**) 128A1030 (Peltron) and (**b**) TK-1-3-S (HTRD) TEMs for different temperature gradient.

The tested TEMs were of two types: 128A1030 (Peltron) and TK-1-3-S (HTRD). Generally, an I-V curve evaluating the power generation performance of the TEM is plotted on each temperature gradient to analyze performance with maximum output (Figure 8 and Table 2). The 128A1030 model and TK-1-3-S have maximum output when external resistance is approximately 1.4 Ω and 2.0 Ω, respectively. Both models have maximum output with external load resistance similar to the internal resistance value. While the railroad vehicle runs, the average temperature difference between the two sides of the TEM is approximately 15 °C, but the TK-1-3-S module has approximately 13% higher output performance. Therefore, the TK-1-3-S module is selected for the field test on a rolling stock with detailed design.

## 4. Results and Discussion

**Figure 9:**
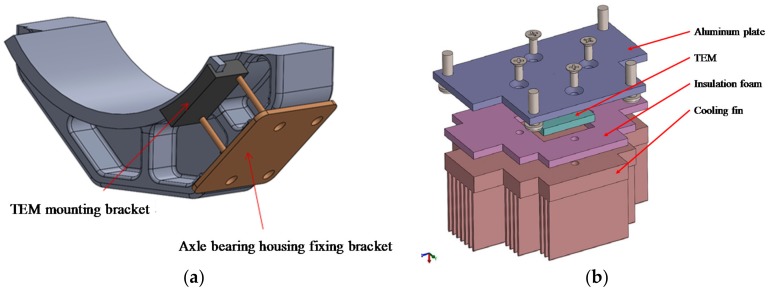
Design of (**a**) TEM mounting and fixing bracket and (**b**) cooling fin for applying on rolling

**Figure 10:**
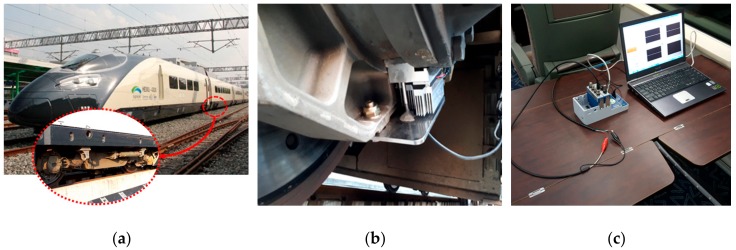
(**a**) HEMU-430X, Korean next generation high speed train, (**b**) TEHS mounted on axle bearing housing of HEMU-430X train, (**c**) On board data measurement setup.

Designed and manufactured for on-board test on a rolling stock based on the performance test of the TEM, a TEHS was installed on the surface of the axle bearing housing of the Korean next-generation high-speed railroad vehicle HEMU-430X (maximum speed 421.3 km/h), a prototype vehicle, between the axle bearing, heat source, and outdoor air. As shown in Figure 9, cooling fins with a TEM were manufactured to minimize the thermal leak of heat transfer from the heat source to the TEM and to maximize the temperature gradient by lowering the temperature of the low-temperature side of the TEM. The final shape of the cooling fins was designed and optimized considering the operation condition of the rolling stock and easy assembly of the protection frame. Figure 10 shows the TEHS with these design conditions applied to the test vehicle.

The power generation test under rolling stock operation was performed. The test track was between Osong and Dongdaegu in Korea, and the maximum driving speed was 300 km/h. The output performance of the TEHS was measured in real time as shown in Figure 10 by a DAQ device (NI 9225, NI, Austin, TX, USA). At the same time, an individual thermocouple was installed on the axle bearing housing to measure the housing surface temperature in real time. 

**Figure 11:**
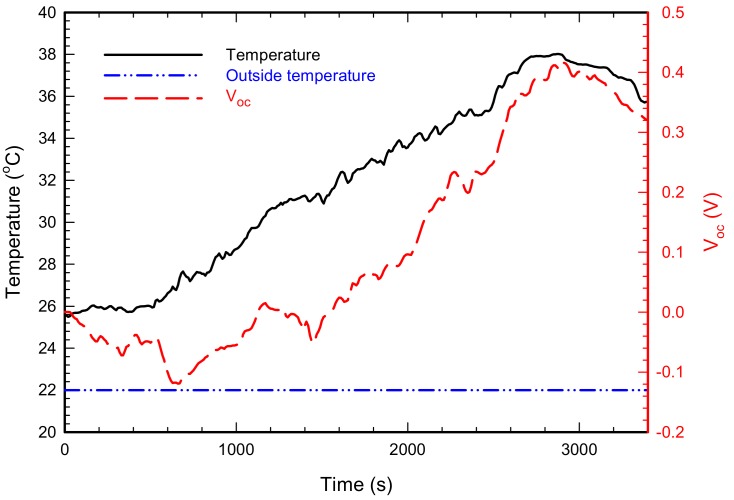
Measurement result of housing surface temperature and output voltage of the TEHS during Osong–Dongdaegu operation.

An open circuit voltage measured at the section between Osong and Dongdaegu is shown in Figure 11 together with the results of housing surface temperature and outdoor temperature. According to the conditions of railroad vehicle operation, the output voltage between the start of driving and approximately 1500 s (approximately 26 min) is unstable. That is because the initial driving speed is low, and the temperature of the axle bearing housing slowly increases, so stabilized power generation by TEM takes time. Actually, the surface temperature of the axle bearing housing increases after 800 s (approximately 13 min), and the maximum surface temperature reaches 38 °C at 2900 s without a temperature decrease. The open circuit voltage rapidly increases after 1600 s, and its maximum voltage is 0.4 V. As with the change in temperature, the output voltage also decreases after 2900 s when the railroad vehicle starts to decelerate to stop in Osong.

**Figure 12:**
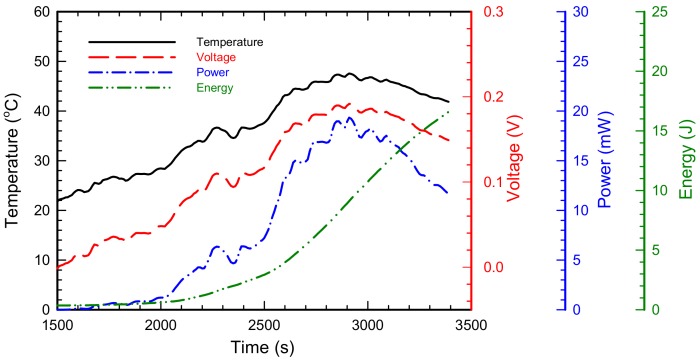
Expected power and energy output of the TEHS during Osong–Dongdaegu operation.

Based on the result of the field test, the estimated performance of the TEHS as a power source is presented in Figure 12. To harvest maximum power from the waste heat, it is important to select the optimal load resistance [37]. The result of the performance test on the TEM in Section 3 suggests that the optimal load resistance varies according to temperature conditions. Note, however, that configuring self-adjusting load resistance in accordance with temperature requires an additional power management circuit, and it may consume unnecessary electric power. Thus, the results in Figure 12 are obtained assuming constant optimal load resistance connected to the TEHS. The average temperature of the housing surface measured by the field test is approximately 36.8 °C, and the average temperature difference on both sides of the TEM is 14.8 °C. Therefore, accepting the result presented in Table 2b, the constant load resistance is assumed to be 1.9 Ω. Along with this condition, the output voltage, electric power, and charging energy with the assumed 100% charging efficiency of the TEHS are as shown in Figure 12. The TEHS has a maximum output voltage of approximately 0.192 V, its maximum power is approximately 19.3 mW, and its charged electric energy after railroad vehicle operation for 3400 s is approximately 16.6 J. Commercial low power sensors usually use 1.8 V or 3.3 V constant voltage source as a power supply. Therefore, the power management circuit is required to regulate power from the TEM, store electrical energy, and finally make constant voltage output. Assuming an electric energy conversion efficiency of 70%, which explains the energy loss of power management, approximately 11.6 J of electric energy can be stored for 3400 s. This amount of energy may continuously operate 30 commercial low-power temperature sensors (1.8 V–30 μA, PCT2202UK, NXP semiconductors, Eindhoven, The Netherland) or continuously operate 2 low-power 3-axis acceleration sensors (3.3 V–0.4 mA, MMA8491Q, NXP semiconductors, Eindhoven, The Netherland) for 3400 s.

## 5. Conclusions

A thermoelectric energy harvesting system storing electrical energy by harvesting waste heat generated by railroad vehicles was designed and manufactured. To maximize the performance during the design process, a performance test on TEMs was performed, and cooling fins were optimized. A TEHS reflecting these optimized results and the compatibility with rolling stocks was manufactured and installed on the axle bearing housing of HEMU-430X, the Korean next-generation high-speed railroad vehicle. The thermoelectric power generation test using the temperature difference between the axle bearing housing and outdoor air during the rolling stock running yields up to 0.4 V of open circuit voltage. Based on the open circuit voltage and temperature data of the field test result, the expected generated power and stored energy can reach a maximum of 19.3 mW and 16.6 J, respectively. These are sufficient for operating a number of commercialized low-power sensors. Note, however, that the generated power is not sufficient at the very beginning of the railroad vehicle operation because it takes some time for the axle bearing housing to increase its temperature. Therefore, in order to use sensor nodes for monitoring the conditions of the running rolling stock in real time, further researches on highly functional, high-efficiency, low-power management are required. By applying the TEM to the rolling stock, the test of converting waste heat into useful electric energy was performed, and the waste heat of railroad vehicles was proven to be usable as a power source. In the future, a number of sensor nodes integrated with TEHS will be installed on various types of railroad vehicles, and the performance will be tested under various operation conditions.

## Figures and Tables

**Table 1:** Temperature difference between heating and cooling parts (°C).

Number of Fin	Height of Fin (mm)					
	31.3	35.3	39.3	43.3	47.3	51.3
7	14.89	15.66	16.34	16.94	17.49	17.97
9	16.12	16.93	17.64	18.25	18.81	19.32
11	16.81	17.65	18.39	19.01	19.57	20.05

**Table 2:** Results of output performance test on TEMs with varying external load resistance, (**a**) results from 128A1030 (Peltron), (**b**) results from TK-1-3-S (HTRD).

**(a)**				
**ΔT (°C)**	**I at P_max_ (mA)**	**V at P_max_ (V)**	**R at P_max_ (Ω)**	**P_max_ (mW)**
10	47.85	0.061	1.278	2.946
15	68.09	0.096	1.406	6.503
20	89.98	0.126	1.403	11.382
25	112.21	0.157	1.401	17.658
30	127.30	0.194	1.524	24.673
**(b)**				
**ΔT (°C)**	**I at P_max_ (mA)**	**V at P_max_ (V)**	**R at P_max_ (Ω)**	**P_max_ (mW)**
10	41.40	0.078	1.892	3.296
15	62.02	0.118	1.900	7.295
20	75.14	0.165	2.197	12.701
25	100.42	0.201	2.001	19.879
30	111.83	0.246	2.197	27.969
